# Extract of a Letter from Mr. Christie, Chief of the Medical Staff, at Ceylon, to Sir Walter Farquhar, Bart

**Published:** 1803-05-01

**Authors:** Thomas Christie

**Affiliations:** Columbo


EXTRACT *of a* LETTER *from* MR. CHRISTIE, *Chief of the Medical Staff, at Ceylon, to* SIR WALTER FARQUHAR, *Bart.*

MY DEAR SIR,

IN a former letter I mentioned to you the failure with the Vaccine matter my brother sent me, by your direction; but I am extremely happy to say, that by successive inoculations at Constantinople, Bagdad, Bussorah, and Bombay, the Cow-Pox has, at length, been introduced into India, and that we have been uncommonly successful in extending it throughout Ceylon, where it will prove the greatest of all possible blessings, as great numbers were annually swept away on this island by that exterminating malady the Small-pox, which was here particularly malignant, and consequently dreaded as the plague.

In addition to the ravages actually committed by the disease itself in the population of the country, whole villages were often destroyed in consequence of its appearance only, as it was by no means uncommon in the more remote parts of the country, for the whole of the inhabitants of a village, to desert their homes on the first appearance of the Small-pox, fly into the jungle, and leave to their fate their unfortunate relations and friends who chanced to be infected; and these, if they escaped the dire attacks of this dreadful distemper, too often fell victims to want, or to their no less relentless enemies the savage wild beasts, which abound in the unfrequented parts of this island.

In September, 1800, I was witness to a most distressing scene of this kind, in the neighbourhood of Ballicaloa, on the eastern side of the island. The Small-pox had broke out suddenly in the village of Enore, about the middle of July, and so great was the panic occasioned amongst the inhabitants, that all those in health immediately deserted their habitations, and left the helpless sick without any assistance whatever.

When I visited the village on the 4th of Sept[illegible] infection had ceased, and the inhabitants were b[illegible] to return to their usual residence, (once a flourish[illegible] lage) but which they now found desolate and wast[illegible] consequence of their precipitate desertion.

Out of thirteen infected persons, six had died, and seve[illegible] remained in a miserable emaciated state. These survivors gave me the following melancholy recital, which was too certainly verified by the appearance of the village.

On the departure of the inhabitants in health, the elephants, spotted tigers, and wild boars immediately came down from the jungle, pulled down the fences, rooted up and destroyed the young trees, ate the stores of rice and other provisions, and what is still more horrible, carried off the sick, or at least consumed the bodies of the deceased; for it is certain, that in one house, where three sick persons had been left, not the least vestige of their remains could be found, on the return of the inhabitants to the village.

With a view to obviate these dreadful calamities, and alleviate as much as possible the horrible consequences of that exterminating malady the Small-pox, Mr. North, with that humanity and liberal policy for which he has ever been distinguished and admired, early in 1800, instituted an establishment for the purpose of affording relief to persons afflicted with this dreadful distemper, preventing the extension of the contagion, and promoting, under certain restrictions, the practice of inoculation.

His Excellency did me the honor to place the whole of this establishment under my immediate superintendance, and during two years which it existed, I have the satisfaction to think, that not only a great many lives were saved by the salatury practice of inoculation, but that the natural disease was rendered much less frequent and malignant.

I am aware of the truth of the observation, that although variolous inoculation has been highly beneficial to individuals, yet it has upon the whole perhaps been hurtful to the community, by keeping up a source of contagion. In the present instance, however, inoculation was strictly prohibited, except in the established hospitals, or in those situations where the contagion was very general throughout a village or district; and even in these cases, the houses in which the Small-pox existed, whether from inoculation or natural infection, were ordered to be placed in a strict state of quarantine, during the continuance of the disease.

That

That much may be done in this way, in preventing the communication of infection, and that inoculation may be practised in well regulated hospitals, without much risk of rendering the contagion general, will appear from the fact, that soon after the establishment of this Institution, the Small-pox was banished from some extensive districts; and that although the contagion was constantly kept up in the Inoculation Hospitals, yet that in some of the districts, particularly Galle and Trincomalée, no case of the natural disease occurred in the surrounding country for several months.

This establisment however could only be considered as alleviating the public calamity, for notwithstanding every attention on the part of the medical men, almost one-third of all the cases of natural Small-pox died, and somewhat more than one in a hundred of our inoculated patients.

It was therefore with the greatest impatience and anxiety, that we looked forward to a more certain remedy for the evil by the introduction of the Cow-pox in India; to promote which desirable event, Mr. North left no measures untried. At length, thank God, from a fortunate succession of inoculations at Constantinople, Bagdad, Bussorah, Bombay, and Trincomalée, we are now in possesion of this inestimable blessing, and the benefits derived from it already on this island have been very great.

The disease was first introduced into Ceylon, by the successful inoculation of John Sybelle at Trincomalée, on the 11th of August; and as soon as success was ascertained in this instance, orders were given by government for the prohibition of variolous inoculation, and for the propagation of the Vaccine disease throughout the different stations on the island by means of inoculated patients.

The first patients were inoculated from John Sybelle on the 20th of August, and since that time so rapid has been our success, that from this source the disease has been extended to every station on the island, and upwards of seven thousand people have been inoculated in less than three months.

As I conceive that every thing relating to the introduction of the Cow-pox into India, and its successful propagation in this remote part of the world, must prove interesting to you, who patronized, in the early stage of its introduction, this most important discovery, which in its consequences must prove infinitely more beneficial to mankind than any which history records, I beg leave to inclose some letters on this subject, which have been published in the different papers in India.

 This

This will be delivered to you by Mr. Carnic, a surgeon on the Madras establishment, who has long done duty on Ceylon, and who has promised to take charge of a cinnamon stick, a Talipot umbrella, and a Ceylon ring, containing a specimen of the different precious stones produced on the island. These trifles are of no intrinsic value, but may possibly be acceptable to you as curiosities.

*Columbo,* I remain, &c.

*Nov.* 19, 1802. THOMAS CHRISTIE.

P. S. The letters from me, addressed to the Governor and to the medical superintendants at Ceylon, were never published; but finding that government have sent them home, as explanatory of the measures adopted for propagating the Cow-pox on this island, I have also enclosed them for your perusal.

